# Developmental Therapeutics for Alveolar Generation and Regeneration: Implications for Bronchopulmonary Dysplasia and Prematurity-Associated Lung Disease

**DOI:** 10.3390/cells15181695

**Published:** 2026-09-18

**Authors:** David Warburton, Rex Moats, Lance A. Parton, Edmund F. LaGamma, Victoria Niklas

**Affiliations:** 1 Division of Newborn Medicine, Maria Fareri Children’s Hospital at Westchester Medical Center, Department of Pediatrics, New York Medical College, Boston Children’s Health Physicians, Valhalla, NY 10595, USAedmund.lagamma@wmchealth.org (E.F.L.); 2 Developmental Biology and Regenerative Medicine Program, Saban Research Institute, Children’s Hospital Los Angeles, Keck School of Medicine, University of Southern California, Los Angeles, CA 90027, USA; 3 Oak Hill Bio Holdings Ltd., Altrincham WA14 2DT, UK; victoria.niklas@oakhillbio.com

**Keywords:** bronchopulmonary dysplasia, developmental therapeutics, extremely preterm infant, fibroblast growth factor 10, insulin-like growth factor-1, lung, regeneration

## Abstract

**Highlights:**

**What are the main findings of the review?**

• The mechanisms of alveolar formation remain incompletely resolved. Classical secondary septation and the proposed epithelial-budding model may represent complementary interpretations of a complex three-dimensional process coordinated by soluble signaling factors and by interactions among epithelial progenitors, pulmonary endothelial cells, mesenchymal and myofibroblast populations, extracellular matrix remodeling, and biomechanical forces.
• Continued postnatal alveolar formation, compensatory lung growth, lineage-tracing studies, and observations of human progenitor cells support the biological plausibility of alveolar repair and regeneration. However, direct demonstration of therapeutically induced regeneration of durable, functional human alveolar-capillary units remains an important unmet challenge.
• Contemporary bronchopulmonary dysplasia (BPD) reflects interrupted alveolar and pulmonary vascular development compounded by postnatal injury. Physiologic insulin-like growth factor 1 (IGF-1) replacement and fibroblast growth factor 10 (FGF10)-mediated signaling illustrate complementary systemic and local developmental strategies for supporting lung maturation after extremely preterm birth.

**What are the implications of the review?**

• These findings may support a broader therapeutic approach in which interventions not only limit postnatal lung injury and inflammation but also seek to preserve or restore the natural developmental signaling required for lung growth and development.
• IGF-1 and FGF10 represent complementary yet unequally advanced therapeutic concepts that may help support natural developmental signaling. Physiologic IGF-1 replacement has progressed through large-animal studies and early clinical evaluation, whereas FGF10-based approaches remain at the preclinical proof-of-concept stage and require further investigation of pulmonary delivery, pharmacology, efficacy, and safety.
• Preliminary therapeutic signals remain hypothesis-generating. Each approach requires independent confirmation in appropriately designed studies that distinguish tissue protection from true structural regeneration and determine whether early developmental effects translate into durable respiratory and neurodevelopmental benefits.

**Abstract:**

The mechanisms by which alveoli form during lung development remain under investigation. The classical model proposes formation through secondary septation, whereas an alternative proposed model suggests that alveoli arise through epithelial budding within preformed mesenchymal stabilizing rings. Regardless of the precise morphogenetic mechanism, alveolar formation depends on coordinated interactions among epithelial progenitor cells, pulmonary endothelial cells, mesenchymal populations, extracellular matrix remodeling, and biomechanical forces. Stereologic imaging supports continued postnatal alveolar formation and the biological plausibility of alveolar regeneration. These observations are relevant to bronchopulmonary dysplasia (BPD), a disorder of interrupted alveolar and pulmonary vascular development after extremely preterm birth. This review integrates competing morphogenetic models with complementary local and systemic therapeutic concepts. Insulin-like growth factor 1 (IGF-1) provides systemic and local support for alveolar, vascular, and multiorgan maturation. In contrast, fibroblast growth factor 10 (FGF10) is a major local morphogen that regulates epithelial branching, alveolar induction, and repair. Physiologic IGF-1 replacement is being evaluated clinically to reduce BPD severity; FGF10-mediated regeneration remains at the preclinical proof-of-concept stage. Together, these pathways illustrate how preserving or resuming developmental signaling may support lifelong lung health after extremely preterm birth.

## 1. Introduction

Bronchopulmonary dysplasia (BPD) is a major contributor to long-term pulmonary morbidity after extremely preterm birth. Impaired alveolarization and microvascular dysgenesis, complicated by secondary postnatal injuries, underlie the BPD pathophysiology [1,2,3,4]. Developing targeted regenerative therapies depends on exploiting well-established mechanisms of distal lung development and endogenous tissue repair [5,6]. Although the precise cellular sequences that drive alveolar morphogenesis are not completely understood, alternative models of secondary septation and epithelial budding suggest a highly coordinated process mediated by multicellular cross-talk, extracellular matrix remodeling, and biomechanical signals [7,8,9,10,11]. The plausibility of restoring lung structure is supported by ongoing postnatal alveologenesis, compensatory lung expansion, and progenitor lineage-tracing approaches [12,13,14,15]. Although restoration of alveolar function through these developmental mechanisms remains a translational challenge, therapeutic strategies are moving toward pro-regenerative approaches designed to protect or restart endogenous morphogenetic cascades [6,16,17,18]. Practical therapeutic options include targeted growth factor interventions, such as systemic physiologic replacement of insulin-like growth factor 1 (IGF-1) and local fibroblast growth factor 10 (FGF10) signaling. These interventions aim to re-establish the essential signaling networks needed for distal lung maturation and functional tissue repair following extremely preterm birth [19,20,21,22]. In this review, we provide an overview of current evidence on the mechanisms of alveolar formation and alveolar generation and regeneration as therapeutic targets, with a focus on developmental therapeutics for BPP and prematurity-associated lung disease.

## 2. Literature Search

This narrative review was informed by searches of PubMed and Google Scholar through August 2026, using combinations of the terms “alveolarization,” “alveologenesis,” “alveolar regeneration,” “bronchopulmonary dysplasia,” “FGF10,” “IGF-1,” “extremely preterm infant,” and “lung development.” Relevant developmental, mechanistic, preclinical, translational, and clinical studies were selected based on their relevance to alveolar and pulmonary vascular development, BPD, and the therapeutic application of FGF10 or IGF-1. Reference lists of key reviews and primary studies were also examined. Relevant studies were selected based on the scope and objectives of the review and did not utilize predefined systematic-review eligibility criteria.

## 3. Extremely Preterm Birth Interrupts Alveolar Formation

Extremely preterm birth, defined as birth before 28 weeks’ gestational age (GA), occurs across the stages of late canalicular and early saccular lung development and generally precedes established alveolarization [5,7,23]. During this period, primitive distal acinar structures transition into thin-walled saccules while epithelial differentiation, surfactant production, formation of the air–blood barrier, and pulmonary microvascular growth remain incomplete. Alveolarization subsequently expands the gas-exchange surface and continues throughout childhood and adolescence, with further lung maturation possibly extending into early adulthood [5,7,12,23]. Across extremely preterm birth, lung maturity varies substantially from 23 to 28 weeks’ GA. Infants born at the earliest GAs have less-developed gas-exchange structures, a thicker air–blood barrier, less mature pulmonary microvasculature, and more limited surfactant production [4,5,7,23]. These factors, together with antenatal factors such as fetal growth restriction, placental dysfunction, inflammation, and infection, may further influence lung maturity and the risk of BPD, defining infants with the highest risk of disease [2,4,24].

Alveolarization depends on coordinated epithelial, endothelial, mesenchymal, and extracellular-matrix interactions [6]. In the developing lung, IGF-1 provides complementary systemic and local support for epithelial, endothelial, and mesenchymal growth, whereas FGF10 acts predominantly as a local mesenchymal signal that regulates epithelial progenitors, morphogenesis, and repair [19,20,25]. After extremely preterm birth, circulating IGF-1 falls below fetal concentrations during a period when saccular and vascular development would normally occur in utero [26,27,28,29]. Although a direct linear interaction has not been established, IGF-1 and FGF10 may functionally converge on epithelial–mesenchymal communication, progenitor activity, angiogenesis, and tissue growth during postnatal lung development [19,20,25].

Advances in neonatal care have increased survival at the earliest GAs and shifted BPD from a predominantly fibroproliferative injury or “old” BPD, to a multifactorial disorder of interrupted alveolar and pulmonary vascular development or “new” BPD [2,3,4]. Because current clinical approaches do not reliably prevent BPD, developmental therapies that restore systemic IGF-1 support or activate local FGF10-dependent regenerative signaling in the lung warrant continued investigation [4,18,21].

## 4. Alveolar Formation: An Unresolved Developmental Question

The mechanisms by which alveoli form during lung development remain a fundamental unanswered question in pulmonary biology. Resolving this question is central to identifying strategies to preserve and restore alveolar development in diseases such as BPD. The classical Weibel–Burri model proposes that alveoli arise through the erection of secondary septa that subdivide pre-existing airspaces [8,23,30,31]. In the septation model, secondary septa appear as finger-like protrusions into the lumen, with α-smooth muscle actin-positive myofibroblasts at their tips; three-dimensional (3D) imaging reported elsewhere shows these myofibroblasts arranged in an interconnected, ring-like “fishnet” pattern underlying the septal ridges [9]. Similarly, epithelial cells undergo sequential clustering, hollowing, and extension during alveolar formation and maturation [10].

**Figure 1:**
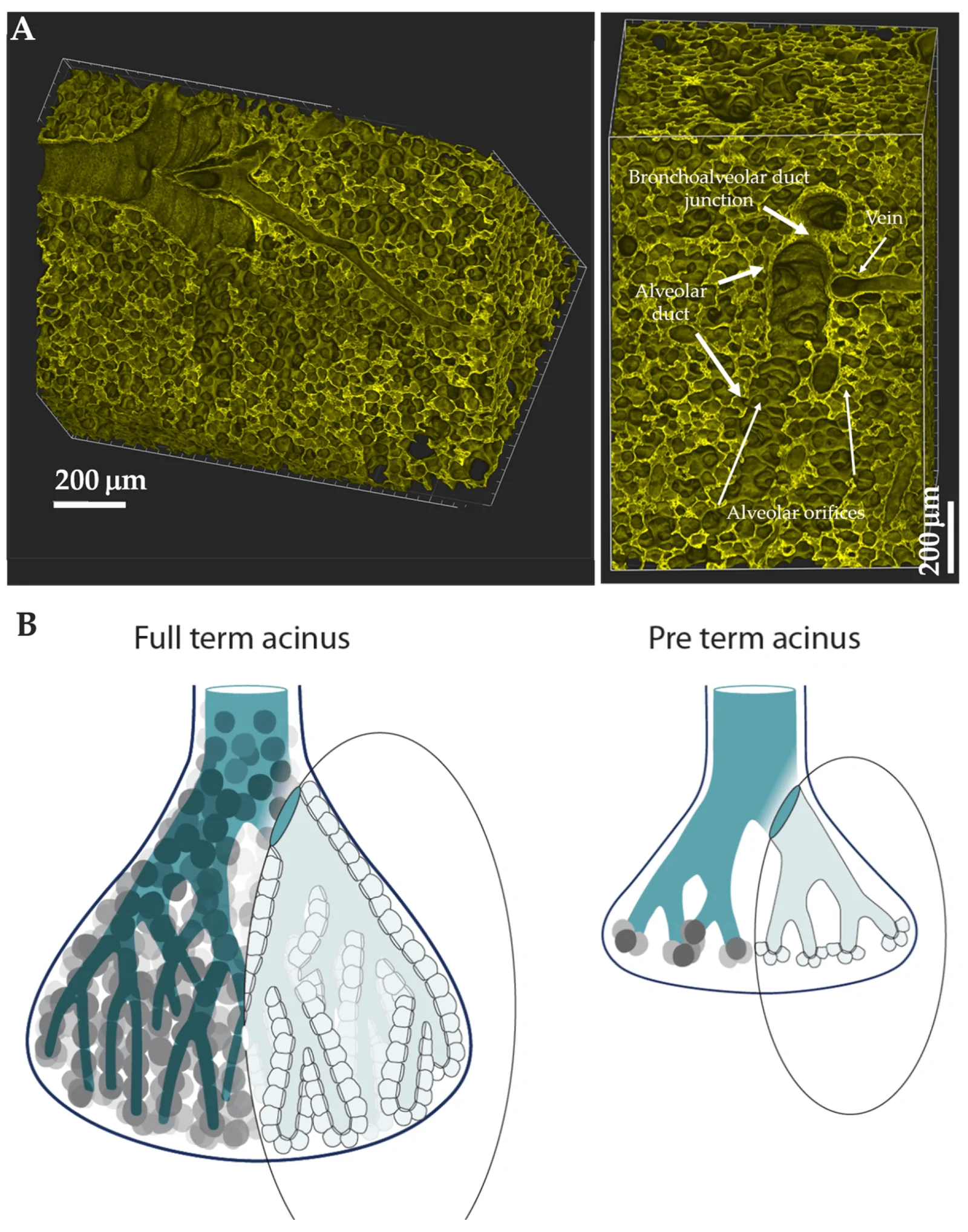
Key alveolar arrangement characteristics. (**A**) 3D reconstructions of the murine lung. Fluorescence microscopy raw images were downloaded from LungMap under CC BY 4.0 license and analyzed using Vibratome serial section imaging microscopy (Vibra-SSIM) technology (reproduced from Warburton et al., 2021 under CC BY 4.0 license) [11,32]. The left and right panels show different planes of a section through the lung. A bronchoalveolar duct, alveolar ducts, and individual orifices can be observed as indicated by the white arrows. (**B**) Schematic visualization of the full-term and preterm acinus to illustrate the proposed alveolar budding model.

More recently, an alternative mechanism has been proposed in which alveoli develop by epithelial budding through preformed mesenchymal stabilizing rings, analogous to epithelial branching morphogenesis during early embryonic lung development [11]. In this proposed model, elastin- and collagen-rich “purse-string” structures may stabilize the alveolar orifice while allowing the expanding alveolar epithelium to protrude into the surrounding compliant mesenchyme [11]. Figure 1 illustrates this 3D organization of the distal acinus and features alveoli that arise from the lateral walls and distal termini of alveolar ducts in contrast with normal full-term and preterm acinar architecture. Most alveoli appear to arise from the lateral walls of alveolar ducts [11].

**Figure 2:**
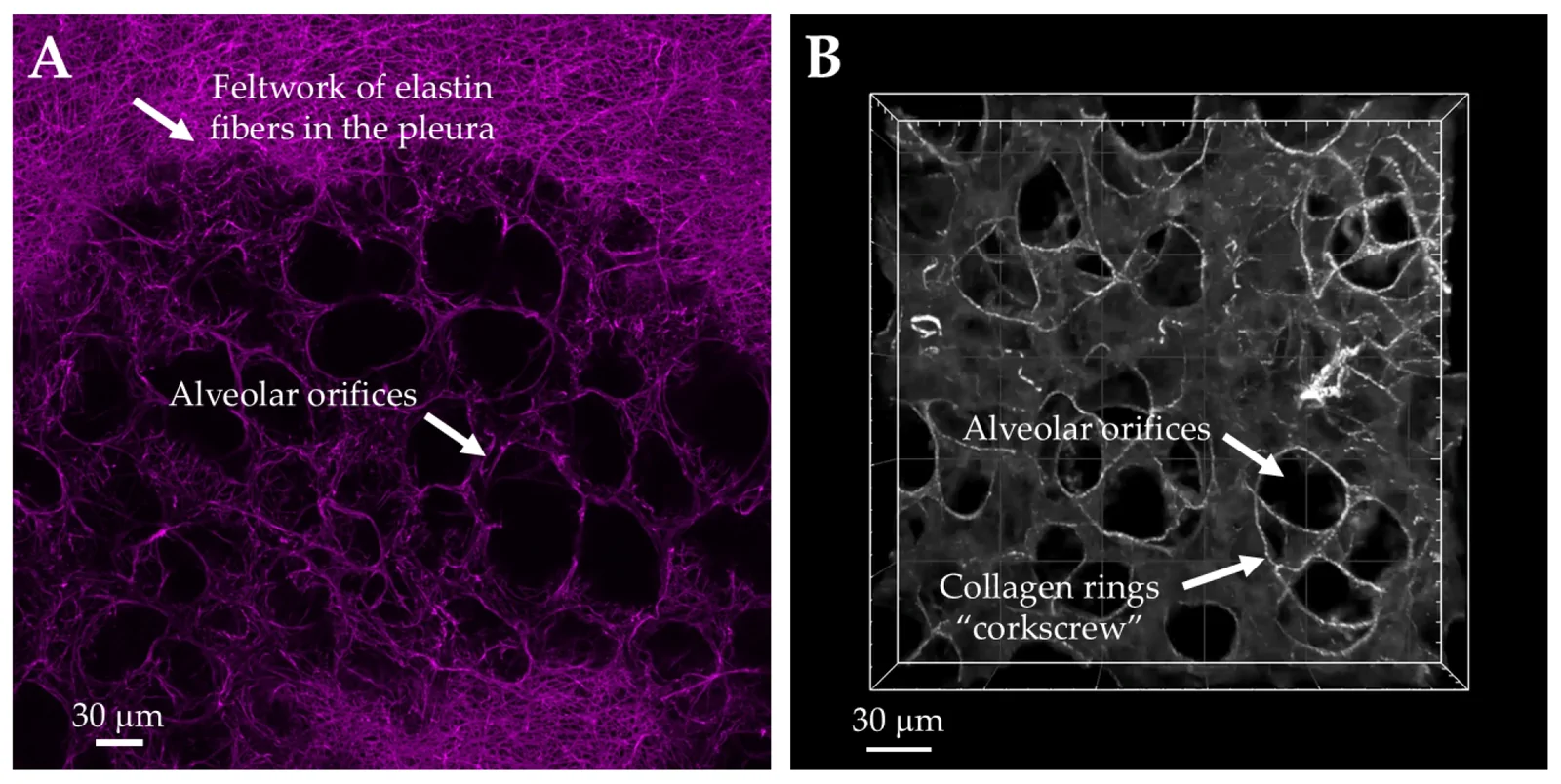
Alveolar os matrix comprises elastin (**A**) and collagen fibers (**B**). (**A**) Elastin fibers (shown in magenta) form a dense feltwork within the lung pleura and are interconnected with the alveolar orifice network, which may contribute to elastic interdependence of the lung and alveolar springiness (Image from LungMap dataset reused under the Creative Commons Attribution [CC BY 4.0 license]) [32]. (**B**) Second harmonic generation (SHG) of collagen fibers (shown in white) located within the lip of each alveolar os. Additionally, corkscrew-like collagen fibers support the alveolar duct, leading to a group of alveolar orifices (reproduced from Warburton et al., 2021 under CC BY 4.0 license) [11].

Because alveolar ducts maintain a relatively constant diameter, these lateral alveoli may be more consistent with a budding mechanism rather than septal subdivision. Similar stabilizing ring structures also appear to surround clusters of alveoli developing at the distal ends of alveolar ducts [11]. Each alveolus communicates with the alveolar duct through a circular or oval entrance, termed the alveolar opening, or alveolar os, which is bounded by a mesenchymal–elastic entrance ring. These stabilizing rings comprise interconnected elastin and collagen fiber networks that surround the alveolar opening and provide structural support for alveolar expansion while maintaining elastic interdependence throughout the peripheral lung (Figure 2). Although the precise morphologic mechanism remains debated, alveolarization is increasingly recognized as a highly coordinated developmental process requiring reciprocal interactions among epithelial progenitor cells, pulmonary endothelial cells, mesenchymal cells, extracellular matrix remodeling, and biomechanical forces.

## 5. Alveolar Regeneration Is Biologically Plausible

Alveolar development continues well beyond normal term birth. However, the expansion of the existing alveoli dimensions alone cannot account for the substantial increase in lung volume during childhood and adolescence. Therefore, it is proposed that new alveoli continue to form throughout somatic growth [13,14,33]. Although children born preterm, including those with BPD, can exhibit continued lung growth and some functional improvement, lung function rarely reaches that of healthy term-born individuals, and long-term reductions in pulmonary reserve and exercise capacity frequently persist [1].

The progressive loss of alveolar architecture characteristic of chronic obstructive pulmonary disease (COPD) and emphysema in older adults shares features with the developmental arrest and alveolar impairment in BPD, including extracellular matrix disruption and impaired vascular development. These similarities provide a conceptual framework for considering shared signaling pathways involved in lung repair and regeneration, while recognizing the important differences in disease origin, timing, and pathophysiology [16,34,35].

**Figure 3:**
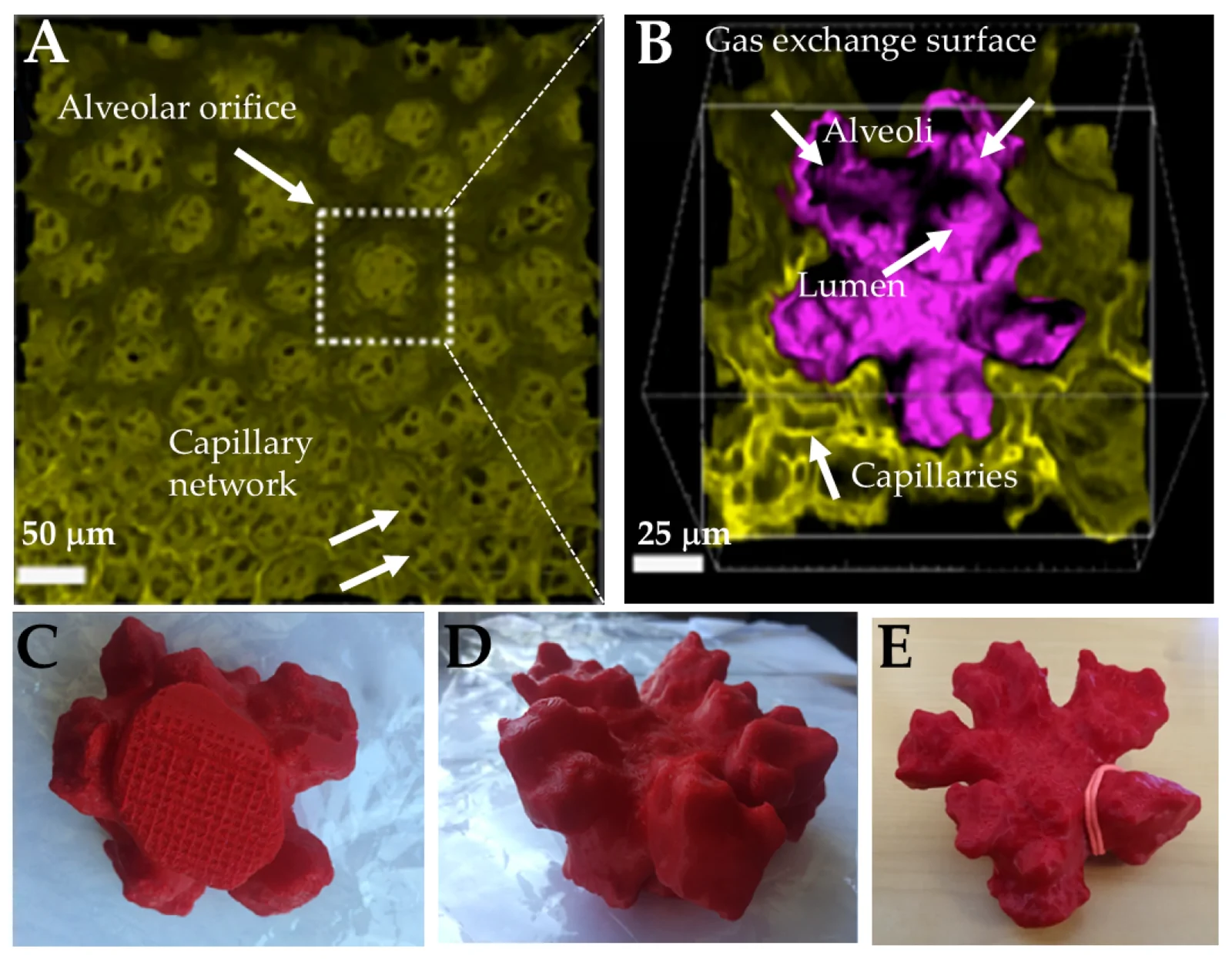
Key features (indicated by white arrows) of peripheral alveoli in a murine lung model at 14 days postnatal age (**A**–**E**) reproduced from Warburton et al., 2021 under the Creative Commons Attribution (CC BY 4.0 license) [11,32]. (**A**) 3D digital reconstruction of peripheral air sacs and subjacent alveolar capillary network within the fluorescent membrane (shown in green). (**B**) Magnified image of the family of air sacs surrounded by the dotted box in (**A**) The gas exchange surface has been digitally rendered in magenta. Typically, 5 individual alveoli, each with an irregular shape and surface rugosity, are connected to a central lumen within the lung mesenchyme, as shown. (**C**–**E**) Complementary views of a 3D solid red object representing a 3D-printed view of the family of air sacs in (**B**) (shown in magenta). (**C**) Proximal to distal view of the alveolar duct. (**D**) Oblique view of the surface of the distal pleural. (**E**) Distal to proximal view of the five interconnected air sacs. An elastic band has been placed around the mouth of one air sac to illustrate the position of the collagen mouth rings shown in Figure 2B relative to the alveolar orifice.

**Figure 4:**
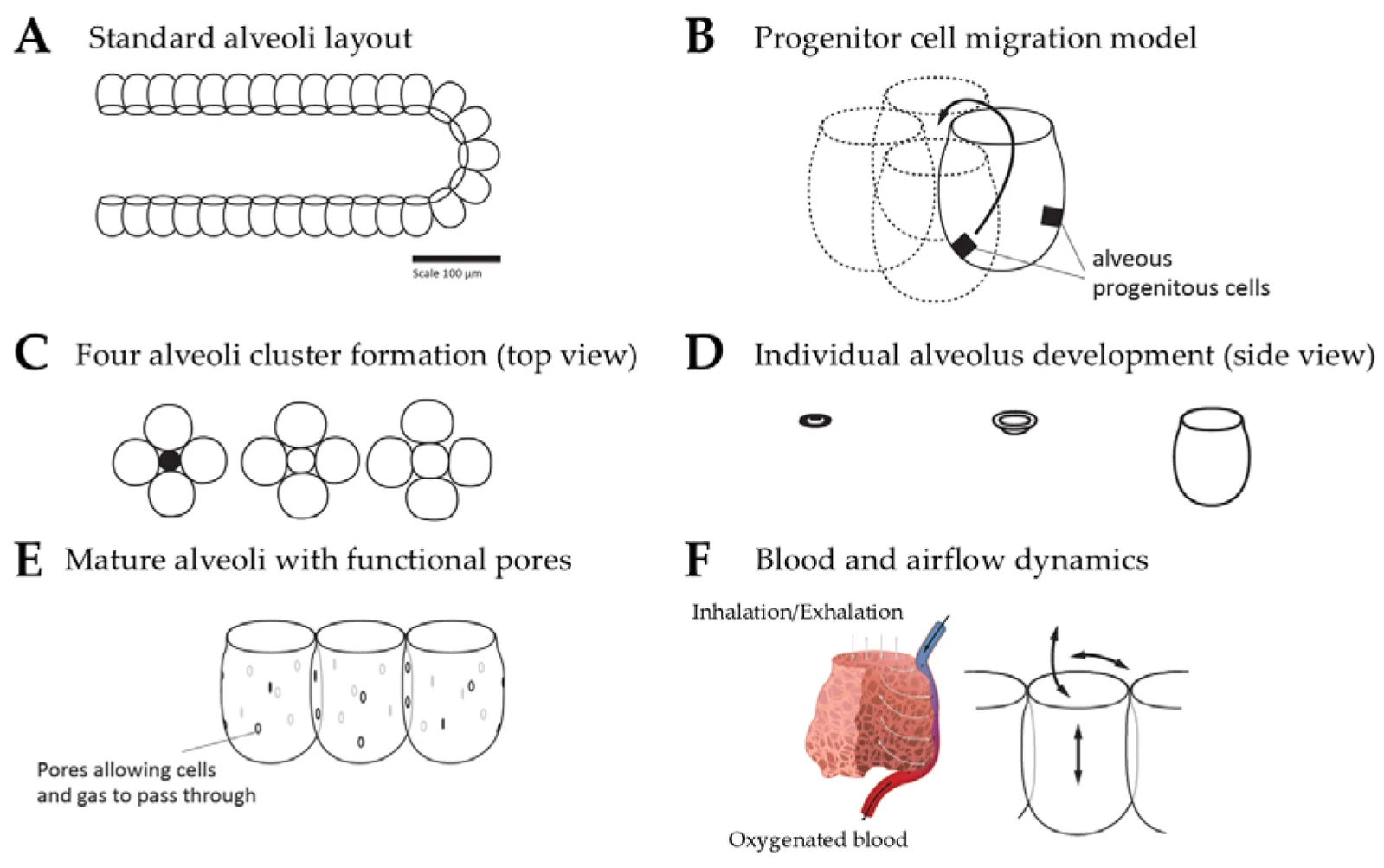
Schematic illustrations of alveoli layout (**A**), cell migration (**B**), and different stages of alveolar generation, development, and maturation (**C**–**E**) and the blood flow and gas exchange across an alveolar wall with a capillary network (**F**).

Figure 3 presents 3D reconstructions of the peripheral alveoli in a murine lung model (Figure 3A,B) and the extracellular matrix architecture (Figure 3C–E). A schematic model of alveolar generation illustrates how developmental mechanisms underlying normal alveolar formation may also provide the biological basis for postnatal regeneration (Figure 4). Indeed, adult survivors of extreme preterm birth frequently exhibit reduced pulmonary reserve and fixed airflow limitation consistent with some characteristics of COPD, emphasizing the lifelong consequences of disrupted alveolar development [1,36].

Although alveolar formation has not yet been observed in real-time microscopy imaging in humans, several observations from stereological studies and gas-diffusion magnetic resonance imaging support postnatal alveolarization and the regenerative capacity of the mature lung across different physiologic conditions. Following pneumonectomy, the lung can undergo compensatory growth and structural remodeling, as evidenced by restoration of gas-exchange capacity in humans [15]. Adaptive remodeling at high altitude requires increased lung diffusing capacity and other changes in pulmonary function to occur in response to chronic hypoxic exposure [13,37]. Cellular regeneration is supported by lineage-tracing studies of alveolar epithelial progenitors, which have demonstrated that defined epithelial progenitor populations can contribute to alveolar regeneration after injury, whereas human studies have identified distal-airway secretory cells capable of generating alveolar epithelial cells [13,33]. Dynamic epithelial cell function during alveologenesis has been successfully reproduced in ex vivo precision-cut lung slices from rodent models [10,38]. Taken together, these findings support the plausibility of the biological alveolar regeneration hypothesis and suggest that therapeutic reactivation of developmental lung morphogenesis after extreme preterm delivery may be feasible and warrants further investigation for the prevention or treatment of alveolar developmental arrest or lung injury.

## 6. Developmental Signaling Networks as Therapeutic Targets

**Figure 5:**
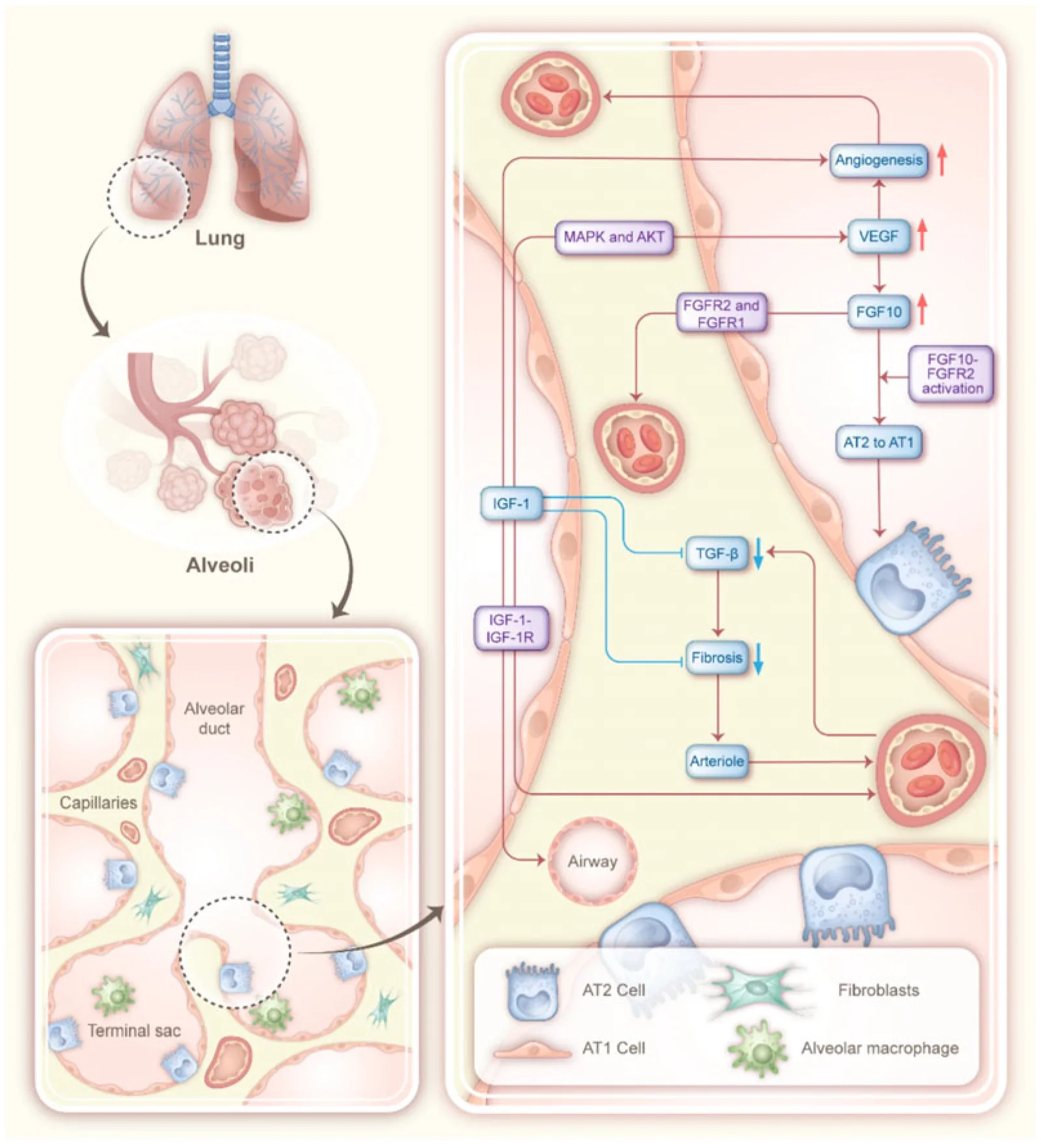
Illustration of the potential central role of insulin-like growth factor-1 in preterm lung development. AT1/2, alveolar type 1/2; IGF-1/R, insulin growth factor-1/receptor; FGF10, fibroblast growth factor 10; FGFR1/2, fibroblast growth factor receptor 1/2; MAPK, mitogen-activated protein kinase; TGF-β, transforming growth factor beta; VEGF, vascular endothelial growth factor. (reproduced from Cui X., 2024 under the Creative Commons Attribution [CC BY 4.0] license) [6].

The capacity for alveolar regeneration likely requires coordinated activation of multiple developmental pathways that govern epithelial progenitor recruitment, mesenchymal remodeling, extracellular matrix organization, pulmonary angiogenesis, and biomechanical regulation (Figure 5) [6].

Within this interconnected network, IGF-1 and FGF10 represent distinct but potentially complementary developmental axes. IGF-1 provides systemic and local support for epithelial, endothelial, and mesenchymal maturation and interacts with angiogenic pathways, including vascular endothelial growth factor (VEGF) signaling. FGF10–Fibroblast Growth Factor Receptor 2b (FGFR2b) signaling supports alveolar type 2 (AT2) specification, proliferation, identity, and survival and may indirectly coordinate alveolar type 1 (AT1) formation; it should not be interpreted as a simple linear pathway of AT2-to-AT1 differentiation. FGF10 is a major local mesenchyme-derived morphogen that regulates epithelial progenitors, AT2-cell development, branching, and repair. Extremely preterm birth may disrupt both axes: placental separation is followed by a reduction in circulating IGF-1, whereas inflammation, hyperoxia, and mechanical ventilation can suppress local FGF10 signaling. These convergent disruptions provide the rationale for investigating local FGF10-mediated regeneration and physiologic IGF-1 replacement as developmentally distinct strategies to reduce BPD risk or severity.

During embryonic development, mesenchyme-derived FGF10 induces the proliferation and outgrowth of lung buds from the foregut endoderm and subsequently coordinates airway branching through interactions with Forkhead Box F1 (FOXF1), T-box Transcription Factor 4 (TBX4), T-box Transcription Factor 5 (TBX5), Wingless-related integration site (Wnt), Sonic Hedgehog Homolog (SHH), and Sprouty Homolog (SPRY) signaling [19,39,40,41,42]. Genetic deletion of either Fgf10 or its principal epithelial receptor, Fgfr2b, fails in lung development distal to the primary bronchial buds, demonstrating the essential role of this pathway in pulmonary morphogenesis [39,40]. FGF10 also contributes to distal lung maturation, AT2-cell development, surfactant expression, alveolarization, and epithelial repair. In neonatal Fgf10-deficient mice, hyperoxia causes more severe hypoalveolarization, impaired AT2-cell differentiation, and reduced surfactant expression [43]. FGF10 deficiency also reduces pulmonary vascular density and increases vascular muscularization, reproducing features of BPD-associated pulmonary hypertension [44]. These findings suggest that FGF10 coordinates epithelial and pulmonary vascular development during alveolar formation.

Premature birth may further compromise this pathway through developmental arrest and postnatal injury and inflammation in response to required care practices after birth. Experimental and human-tissue studies indicate that inflammatory mediators, including Nuclear Factor Kappa Light Chain Enhancer of Activated B Cells (NF-κB), tumor necrosis factor-α, and interleukin-1β, as well as hyperoxic exposure, can reduce or disrupt FGF10 expression [19,45,46]. Variants affecting FGF10 signaling have also been associated with impaired alveolarization, pulmonary vascular remodeling, and susceptibility to BPD in preterm infants [22,46]. Related variants have been linked to COPD in adult smokers, suggesting that reduced FGF10 signaling may establish a developmental susceptibility that becomes clinically apparent after environmental injury [47]. FGF10 is produced predominantly by lung mesenchymal cells, including progenitors that contribute to the lipofibroblast lineage during alveologenesis [19,48,49,50]. Reduced FGF10 production may therefore reflect dysfunction of the mesenchymal niche. Alternatively, impaired alveolarization may arise from abnormalities in the responding epithelium or FGFR2b signaling, potentially limiting the effectiveness of exogenous FGF10 [42,51,52,53].

Recombinant FGF10 has attenuated particulate matter– and lipopolysaccharide-induced inflammatory lung injury, promoted recovery after smoke inhalation, reduced elastase-induced emphysematous injury, and supported structural alveolar regeneration after neonatal hyperoxic injury [22,53,54,55,56]. These findings establish the biological plausibility of FGF10-mediated lung repair but are based on models with different anatomical compartments, mechanisms of injury, and treatment windows. Reductions in acute inflammation or epithelial injury should therefore be distinguished from the coordinated reconstruction of functional alveolar–capillary units. Delivery and safety also require further investigation. Systemic intravenous (IV) FGF10 delivery has been associated with off-target effects, including tongue and eyelid swelling and increased intestinal mucus production, suggesting that localized pulmonary delivery may provide a more targeted therapeutic approach with reduced systemic exposure [57]. In addition, broad systemic exposure raises safety concerns about off-target binding to ectopic targets across other epithelial tissues that express FGFR2b, and the potential risks of uncontrolled epithelial hyperproliferation, disrupted tissue remodeling, or tumor promotion caused by sustained FGF10 signaling [52,57]. Together, these results indicate that localized pulmonary administration could offer a more targeted therapeutic strategy while limiting systemic exposure. Consequently, recombinant FGF10 remains a promising preclinical candidate, but formal pharmacologic, delivery, efficacy, and safety studies (e.g., localized toxicity assessment; long-term proliferative safety) are needed before clinical evaluation in extremely preterm infants [43,44].

The abrupt loss of physiologic IGF-1 after extremely preterm birth represents a potentially modifiable contributor to disrupted alveolar and pulmonary microvascular development, providing the rationale for evaluating IGF-1 replacement using mechanistic, preclinical, and early-stage clinical studies. During the third trimester, circulating IGF-1 concentrations increase in parallel with fetal growth and lung maturation. After extremely preterm birth, concentrations fall rapidly and remain substantially below corresponding intrauterine levels until endogenous production increases several weeks later [26,27]. This nadir coincides with the developmental period when alveolar formation and pulmonary microvascular expansion are normally active. The immature lung therefore experiences a “double hit”: withdrawal of an important systemic developmental signal combined with exposure to oxygen, mechanical ventilation, inflammation, and infection.

Low early postnatal IGF-1 concentrations may precede and be associated with subsequent BPD development [26,27,29,58]. Although observational associations do not establish causality, they are supported by experimental evidence. Disruption of IGF-1 or IGF-1R signaling leads to simplified distal airspaces, abnormal extracellular matrix organization, impaired pulmonary vascular development, and defective alveolar morphogenesis [25,59,60]. Conversely, recombinant IGF-1 promotes morphogenesis in fetal lung explants, supporting its role as a developmental regulator rather than simply a biomarker or permissive growth factor [59]. These effects extend to the pulmonary vasculature. Antenatal injury models in rodents, including preeclampsia and chorioamnionitis, suppress vascular growth and VEGF signaling and reproduce features of contemporary BPD [61,62,63]. Pulmonary endothelial cells also provide angiocrine signals that regulate epithelial and alveolar development through pathways involving nitric oxide, hepatocyte growth factor, vitamin A, VEGF, and IGF-1 [62,64]. Systemic IGF-1 deficiency may therefore impair pulmonary microvascular expansion, constrain alveolar development, and contribute to pulmonary hypertension.

These findings support physiologic IGF-1 replacement rather than supraphysiologic pharmacologic stimulation. Continuous replacement is intended to restore the developmental environment that was withdrawn after premature birth and to maintain circulating IGF-1 within the corresponding intrauterine range. In mechanically ventilated preterm lambs with evolving BPD, continuous IGF-1 replacement restored circulating concentrations toward physiologic levels and improved indices of alveolarization, pulmonary capillary growth, gas exchange, lung mechanics, and pulmonary vascular remodeling despite ongoing respiratory support and oxygen exposure [64,65]. These results provide translational evidence in the lamb model of BPD that restoration of physiologic IGF-1 exposure may preserve coordinated alveolar–vascular development after preterm birth.

IGF-1 and FGF10 should therefore be viewed as complementary rather than competing therapeutic concepts. Physiologic IGF-1 replacement seeks to re-establish the systemic developmental environment that supports coordinated epithelial, endothelial, and mesenchymal maturation. Restoration of IGF-1 may also permit endogenous local pathways, including FGF10- and VEGF-mediated signaling, to operate during the critical developmental period after extremely preterm birth. FGF10-based treatment seeks to restore local morphogenetic and regenerative signaling within epithelial–mesenchymal niches. Together, these approaches illustrate the broader concept of developmental therapeutics: preserving or resuming developmental processes interrupted by premature birth rather than acting solely on established injury or inflammation. Table 1 summarizes the mechanistic, preclinical, translational, and clinical evidence supporting IGF-1 and FGF10 as potential strategies to reduce the risk or severity of BPD and improve long-term respiratory health.

## 7. Pharmacologic Rationale and Clinical Translation of Physiologic IGF-1 Replacement

Physiologic IGF-1 replacement is intended to restore a developmental signal that was abruptly withdrawn after extremely preterm birth, rather than to stimulate growth through supraphysiologic exposure. In fetal life, continuous placental support helps maintain circulating IGF-1. In older children and adults, IGF-1 circulates predominantly in a ternary complex with insulin-like growth factor-binding protein 3 (IGFBP-3) and acid-labile subunit, extending its circulating half-life to more than 12 h [73]. Extremely preterm infants produce little acid-labile subunit and cannot efficiently form this stable ternary complex, contributing to rapid IGF-1 clearance and prolonged postnatal deficiency [63,74,75].

This developmental physiology guided the design of mecasermin rinfabate, a recombinant human IGF-1/IGFBP-3 binary complex. Complexing IGF-1 with IGFBP-3 limits free IGF-1 exposure and may reduce off-target activation of the insulin receptor. Nevertheless, the circulating half-life remains approximately 1 h in extremely preterm infants; continuous IV infusion is therefore required to maintain concentrations within gestational-age–appropriate physiologic ranges during the critical period of alveolar and pulmonary vascular development [28,63]. Therapeutic success depends on achieving physiologic exposure rather than on administering higher doses. Circulating concentrations can be measured using bedside lateral-flow assays or standardized central-laboratory enzyme-linked immunosorbent assays, allowing exposure-guided dose adjustment. This combination of fetal and postnatal reference ranges provides a practical framework for precision developmental therapy.

The preterm-lamb findings summarized above provide a translational bridge from developmental biology to clinical pharmacology. Their exposure-response pattern supports restoration of physiologic signaling, rather than supraphysiologic stimulation, as the proposed therapeutic mechanism [64,65].

An early Phase 2a randomized trial evaluated continuous IV rhIGF-1/rhIGFBP-3 (OHB-607) replacement in 121 extremely preterm infants (n = 61 in the OHB-607 arm versus n = 60 in the standard neonatal care arm). The study did not meet its primary endpoint of reducing the occurrence or severity of retinopathy of prematurity. There was, however, a 53% decrease in severe BPD in the full analysis set (21.3% treated vs. 44.9% standard care) and an 89% decrease in the evaluable set (meeting target physiologic levels) (4.8% vs. 44.9%; *p* = 0.04 and *p* = 0.02, respectively) for severity distribution between groups. There was also a nonsignificant trend toward a decrease in grades 3–4 intraventricular hemorrhage in the full analysis set (13.1% vs. 23.3%) and in the evaluable set (8.3% vs. 23.3%). Fatal serious adverse events occurred in 12 of 61 treated infants and 7 of 60 control infants, although none was considered treatment-related by the investigators or an independent data safety monitoring board [28] (Clinical Trial Identifier NCT01096784).

The results of the Phase 2a study provided a preliminary indication of pulmonary signal and demonstrated the feasibility of continuous IGF-1 replacement using OHB-607, leading to the ongoing Phase 2b study, which has completed enrollment and is expected to report results in the near future [21,28,71,72] (Clinical Trial Identifier NCT03253263). This study is evaluating the impact of OHB-607 on the reduction in severe BPD measured at 36 weeks’ postmenstrual age [3,4,76,77,78]. Accordingly, this study, along with other clinical trials in BPD, incorporates severity-based outcomes using the modified National Institute of Child Health and Human Development and Jensen classifications [3,76,77,78] to assess clinical benefit in BPD, alongside robust safety monitoring for metabolic, neurologic, cardiac, ophthalmologic, and other systemic effects [71].

Clinical translation presents additional practical challenges. Continuous replacement requires prolonged IV access and exposure-guided dosing to maintain gestational age–appropriate concentrations [18,28,71,79]. Rigorous neonatal central line care bundles are essential for reducing catheter-associated complications [71,80]. Early clinical tolerability has been manageable, but the incidence and severity of longer-term adverse effects—including hypoglycemia, other metabolic disturbances, pathologic tissue growth, and potential proliferative risks—remain to be determined [28,64,65,81]. These considerations are being addressed through continued clinical evaluation of physiologic IGF-1 replacement for severe BPD and other complications of extreme prematurity under supervision by an independent data safety monitoring board.

## 8. Summary and Future Directions: From Alveolar Morphogenesis to Developmental Therapeutics

The mechanisms by which alveoli form remain incompletely resolved. The classical secondary-septation model and the proposed epithelial-budding model offer different interpretations of distal lung morphogenesis and may capture different aspects or stages of the same 3D process. In particular, two-dimensional septal ridges may represent sections through elastin- and collagen-rich alveolar entrance rings. Determining how these rings, epithelial progenitors, myofibroblasts, pulmonary endothelial cells, extracellular matrix remodeling, and biomechanical forces interact is essential for understanding both normal alveolar generation and its regeneration following disruption in BPD.

Evidence that alveolar formation continues after birth and during somatic growth suggests substantial developmental plasticity. Compensatory growth after pneumonectomy, adaptation to chronic hypoxia, lineage-tracing studies, human distal-airway progenitor observations, and ex vivo lung models further support the biological plausibility of alveolar repair and regeneration [10,13,15,33,37,82]. These phenomena should not, however, be treated as synonymous. Continued developmental alveolarization, compensatory enlargement, repair after injury, and reconstruction of complete functional alveolar–capillary units are related but distinct processes. Direct demonstration of therapeutically induced regeneration of durable human alveolar–capillary units remains an important unmet objective of these investigations.

This distinction is especially relevant to BPD. Extremely preterm birth interrupts saccular, alveolar, and pulmonary vascular development before the gas-exchange surface has matured. Oxygen exposure, mechanical ventilation, inflammation, infection, and even preexisting placental disease or impaired fetal growth also impact disease pathogenesis in the immature lung. Contemporary, or “new” BPD, is thought to reflect the interaction between interrupted morphogenesis and superimposed injury rather than just injury alone. The notion of developmental therapeutics is intended to complement established neonatal care by preserving or resuming developmental processes interrupted by premature birth.

IGF-1 and FGF10 illustrate complementary but unequally advanced therapeutic concepts. FGF10 is a local mesenchymal morphogen with mechanistic and rodent proof-of-concept evidence for epithelial support and alveolar repair, but it lacks a comparable premature large-animal or first-in-human program establishing delivery, pharmacology, dose, efficacy, and safety. Physiologic IGF-1 replacement seeks to restore a systemic developmental deficiency and has progressed through large-animal studies and early clinical evaluation. Its preliminary pulmonary signal remains hypothesis-generating and requires confirmation in adequately powered trials with long-term safety follow-up [21,28,71,72]. Clinical validation of IGF-1 would not establish the efficacy of FGF10 or any other growth factor; each pathway requires independent evaluation.

Future research should combine improved 3D and real-time models of alveolar morphogenesis with human-relevant organoid, precision-cut lung slice, and large-animal systems. Priorities include resolving the relationship between septation and budding, defining how alveolar entrance rings are formed and remodeled, determining how endothelial and mesenchymal niches regulate epithelial fate, and distinguishing tissue protection from true structural regeneration. Translational priorities include establishing local FGF10 delivery and safety, confirming the efficacy and multisystem safety of physiologic IGF-1 replacement, identifying infants most likely to benefit, and determining whether early structural effects produce durable respiratory and neurodevelopmental benefit.

Developmental therapeutics are intended to complement established approaches that may reduce BPD risk or severity, including lung-protective respiratory care, caffeine, vitamin A supplementation, selected postnatal corticosteroid strategies, optimized nutrition, and infection prevention. Emerging approaches involving mesenchymal stromal cells, extracellular vesicles, recombinant surfactant protein D, and vascular-targeted therapies should also be evaluated within this broader developmental framework [17,83,84,85,86,87,88,89].

## Figures and Tables

**Table 1:** Translational evidence for IGF-1 and FGF10 signaling pathways in lung development and BPD. BPD, bronchopulmonary dysplasia; FGF10, fibroblast growth factor 10; IV, intravenous; IVH, intraventricular hemorrhage; rhIGF-1, recombinant human insulin-like growth factor 1; ROP, retinopathy of prematurity.

Evidence Level	IGF-1	FGF10	Translational Significance
Mouse developmental and BPD models	Genetic loss of IGF-1 or IGF-1R signaling impairs distal lung morphogenesis, epithelial differentiation, alveolarization, and pulmonary vascular development. Pharmacologic replacement of IGF-1 preserved alveolar and microvascular structure of the lung [25,66,67]	Reduced Fgf10/Fgfr2b signaling impaired epithelial progenitor maintenance, AT2-cell maturation, surfactant-associated development, alveolarization, and pulmonary vascular formation. Recombinant FGF10 restored mesenchymal niche activity and induced de novo alveologenesis after hyperoxic injury [22,43,44,46,47,53,54,55,68,69]	Mouse studies establish biologic plausibility for both pathways. IGF-1 acts as a systemic and local developmental regulator; FGF10 acts predominantly as a local mesenchymal-to-epithelial morphogen and regenerative signal
Large-animal models	In premature lambs, continuous IV rhIGF-1/rhIGFBP-3 replacement during postnatal IGF-1 deficiency was associated with improved lung compliance, gas exchange, alveolarization, pulmonary capillary development, and reduced pulmonary hypertension [64,65]	A comparable premature large-animal program demonstrating FGF10 pharmacology, delivery, efficacy, and safety has not yet been reported	Premature-lamb studies provide an important bridge between rodent IGF-1 biology and clinical evaluation. The absence of comparable FGF10 large-animal evidence remains a major translational gap
Observational clinical data	Circulating IGF-1 concentrations fall markedly after extremely preterm birth and frequently remain below fetal concentrations during ongoing saccular and pulmonary vascular development. Lower postnatal IGF-1 levels have been associated with impaired growth and greater risk or severity of common complications of prematurity such as ROP, IVH and BPD [26,29]	Lower umbilical-cord-blood FGF10 concentrations have been associated with the development and increasing severity of BPD in preterm infants [70]	Although causality cannot be determined, human data have shown an association between BPD and reduced availability of either IGF-1 or FGF10
Early randomized clinical evidence	In a Phase 2 randomized trial, continuous IV rhIGF-1/rhIGFBP-3 infusion did not meet the primary ROP endpoint. A prespecified secondary analysis showed less severe BPD among treated infants, although interpretation was limited by sample size and incomplete target exposure [28]	No interventional clinical trial of FGF10 for BPD has been reported	IGF-1 has human safety, exposure, and preliminary pulmonary efficacy data. FGF10 remains at the preclinical proof-of-concept stage
Advanced clinical development	A multicenter randomized Phase 2b study is evaluating physiologic rhIGF-1/rhIGFBP-3 replacement for reduction in severe BPD and other complications in infants born at 23^+0/7^–27^+6/7^ weeks’ GA [71,72]	No advanced clinical development program for FGF10 in extremely preterm infants has been reported	IGF-1 has progressed to indication-focused clinical evaluation for severe BPD. FGF10 requires additional large-animal, safety, dose-ranging, and early-phase clinical studies before comparable development might be feasible

## Data Availability

No new data were created or analyzed in this study. Microscopy images downloaded from the publicly available LungMAP datasets have been reused in this article under the CC BY 4.0 license. These data are available online: https://www.lungmap.net/ (accessed on 15 July 2026). The LungMAP consortium and the LungMAP Data Coordinating Center (U24-HL148865) are funded by the National Heart, Lung, and Blood Institute (NHLBI).
